# Supplementary material for: Technology Invention and Mechanism Analysis of Rapid Rooting of Taxus × media Rehder Branches Induced by Agrobacterium rhizogenes
Source: Int J Mol Sci. 2023 Dec 27;25(1):375. doi: 10.3390/ijms25010375 (PMC10779043; doi:10.3390/ijms25010375)
Supplement: Supplementary file 1 [file ijms-25-00375-s001.zip › Fig. S1. - S5..pdf]

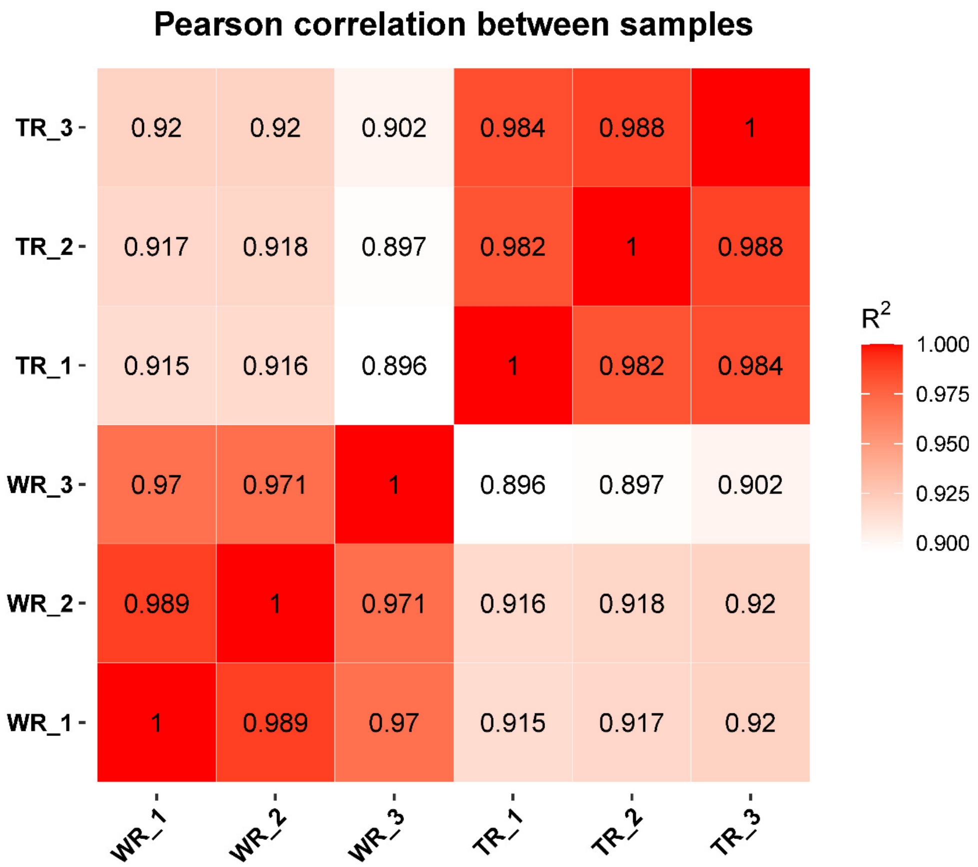


Figure S1. The pearson correlation between samples was over 0.8, which showed that the RNA-Seq data were credible.

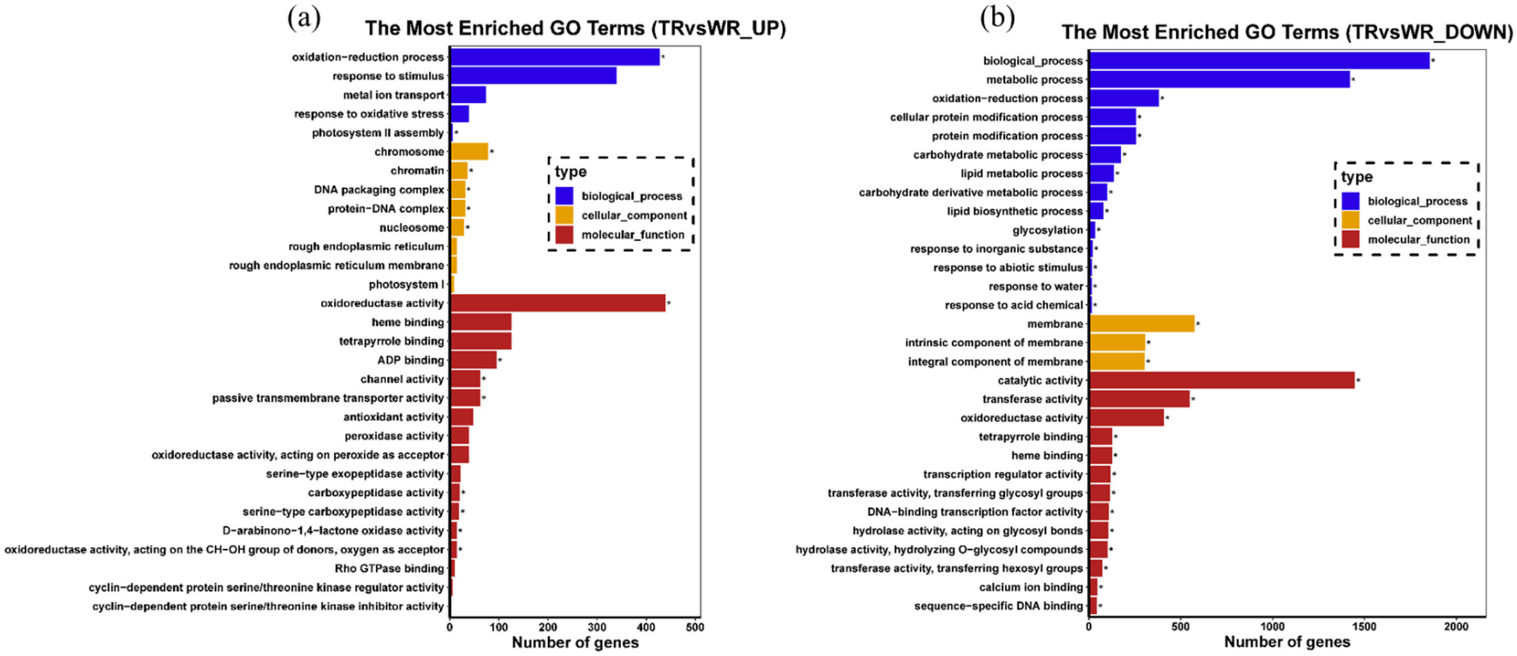


Figure S2. The most enriched GO terms in the upregulated (a) and downregulated (b) GO terms.

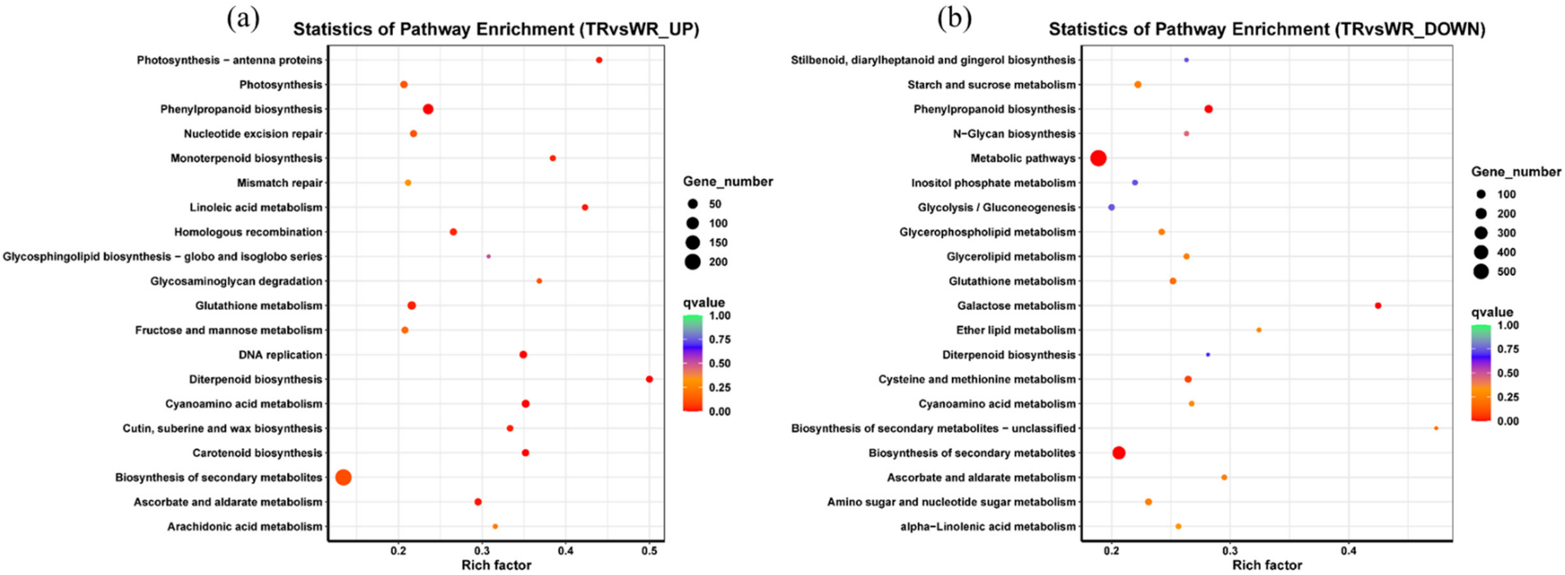


Figure S3. The most enriched KEGG pathways in the upregulated (a) and downregulated (b) KEGG pathways.

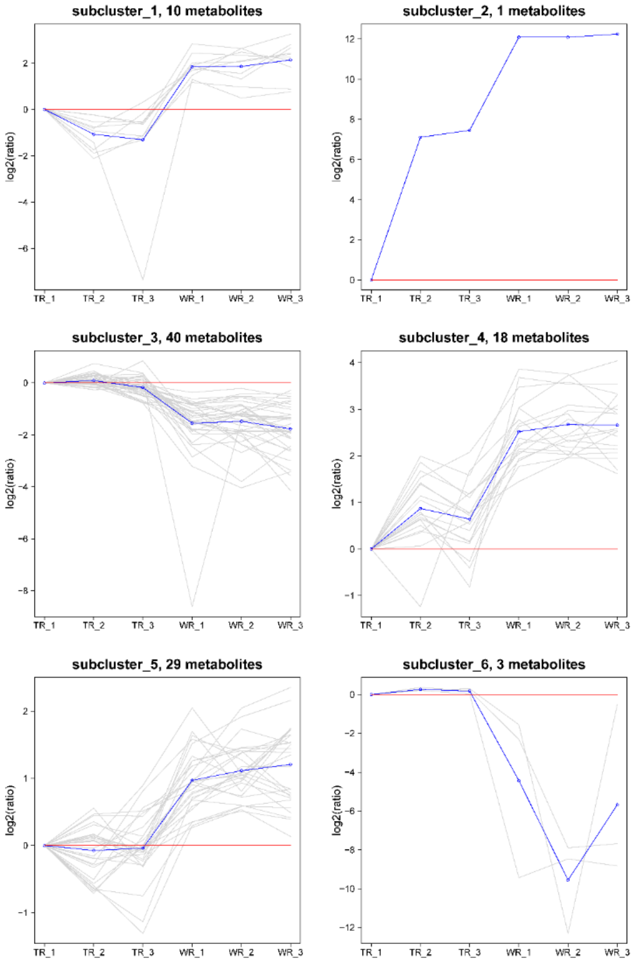


Figure S4. K-means clustering analysis suggested that DEMs might be subdivided into six levels according to the trend in content change of each treatment.

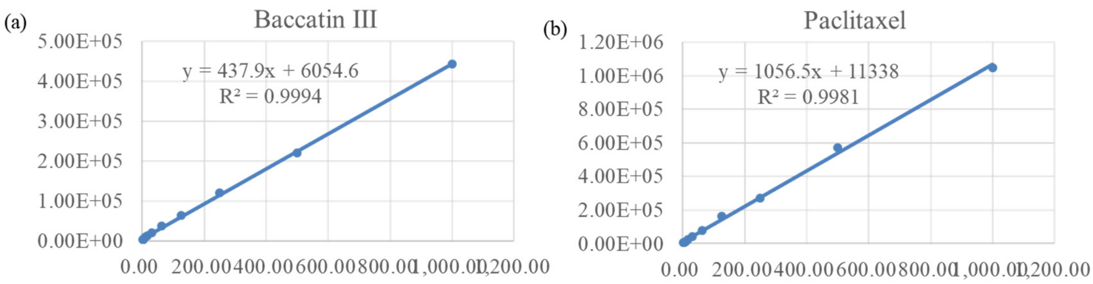


Figure S5. The standard curve of paclitaxel and baccatin III.
