# Supplementary material for: Technology Invention and Mechanism Analysis of Rapid Rooting of Taxus × media Rehder Branches Induced by Agrobacterium rhizogenes
Source: Int J Mol Sci. 2023 Dec 27;25(1):375. doi: 10.3390/ijms25010375 (PMC10779043; doi:10.3390/ijms25010375)
Supplement: Supplementary file 1 [file ijms-25-00375-s001.zip › Table S1. The overall information of sequencing data and the mapping rates of clean reads to reference genome.pdf]

Title: Technology Invention and Mechanism Analysis of Rapid Rooting of *Taxus media* Branches Induced by *Agrobacterium rhizogenes*

Authors: Ying Wang, Xiumei Luo, Haotian Su, Ge Guan, Shuang Liu, Maozhi Ren

Table S1. The overall information of sequencing data and the mapping rates of clean reads to reference genome.

**Sample name**	**WR_1**	**WR_2**	**WR_3**	**TR_1**	**TR_2**	**TR_3**
Total reads	45157926	47208350	41808328	45319254	46702014	49204398
GC content	45.16%	45.17%	47.63%	45.02%	44.94%	44.94%
Q20	98.31%	98.41%	98.67%	98.41%	98.55%	98.44%
Total mapped	40291138 (89.22%)	42254496 (89.51%)	36430630 (87.14%)	40884100 (90.21%)	42086334 (90.12%)	44091726 (89.61%)
